# Efficacy and safety of oral alitretinoin versus oral azathioprine in patients with severe chronic hand eczema: Results from a prematurely discontinued randomized controlled trial

**DOI:** 10.1111/cod.14161

**Published:** 2022-06-01

**Authors:** Angelique N. Voorberg, Esmé Kamphuis, Wietske A. Christoffers, Geertruida L. E. Romeijn, Jart A. F. Oosterhaven, Marie L. A. Schuttelaar

**Affiliations:** ^1^ Department of Dermatology University Medical Center Groningen, University of Groningen Groningen The Netherlands; ^2^ Department of Dermatology Isala Dermatologic Center Zwolle The Netherlands

**Keywords:** alitretinoin, azathioprine, chronic, clinical trial, hand eczema

Alitretinoin is the only registered systemic treatment of severe chronic hand eczema (HE), but it is most effective in hyperkeratotic HE.^1^ It has never been compared in head‐to‐head studies to other immunomodulating systemic drugs, for example, azathioprine, that can be used (off‐label) for severe HE.

In one daily practice case series, patients showed moderate‐to‐good improvement of their HE after treatment with azathioprine.^2^ Based on this case series, in which only one patient with hyperkeratotic HE was included, combined with observations in clinical practice, azathioprine could prove superior to alitretinoin in HE subtypes other than hyperkeratotic HE.

This trial aimed to compare alitretinoin to azathioprine in the treatment of severe chronic HE. Due to a high‐drop out ratio, the trial was unfortunately prematurely discontinued. Most prematurely discontinued studies remain unpublished, resulting in unwanted publication bias. Therefore, we decided to analyse our data.

## METHODS

This was a randomized, open‐label trial in adult patients with severe chronic HE. Hyperkeratotic HE was not included since alitretinoin has proved itself effective in hyperkeratotic HE.^1^ Patients received alitretinoin 30 mg/day or azathioprine 1.5 or 2.5 mg/kg/day for 24 weeks (1:1 randomization). The response was defined as at least two steps of improvement on the photographic guide.^3^ Secondary endpoints included improvement in the Hand Eczema Severity Index (HECSI) score,^4^ the Patient Global Assessment (PaGA)^1^ and the Quality Of Life in Hand Eczema Questionnaire (QOLHEQ).^5^ A full description of the methods can be found in Supporting Information Material S1).

The statistics methods can be found in Supporting Information Material S1. For the sample size, we calculated 58 patients per treatment group. The full sample size calculation can be found in Supporting Information Material S2.

## RESULTS

In total, 42 patients (21 per group) were included. There were no significant differences in baseline characteristics between both groups (see Table S1). The study was prematurely discontinued due to the high dropout rate of 50.0% (*n* = 21/42). The dropout rate was the highest in the azathioprine group with 66.7% (*n* = 14/21) versus 33.3% (*n* = 7/21) in the alitretinoin group.

At the 24 weeks visit, the proportion of responders based on the photographic guide was 64.3% in the alitretinoin group and 14.3% in the azathioprine group (*p* = 0.063). In the alitretinoin group, 57.1% had achieved ‘clear/almost clear’, compared to 14.3% in the azathioprine group (*p* = 0.032). Subjects in the alitretinoin group had a mean decrease of 69.1% of the HECSI score, compared to a decrease of 55.0% in the azathioprine group (see Figure S1A). For patient assessed severity, using the PaGA, 33.3% of the subjects in the alitretinoin group reported ‘clear/almost clear’ at 24 weeks compared to 20.0% in the azathioprine group (*p* = 0.730). Lastly, it is important to note that in three patients in the alitretinoin group, and two patients in the azathioprine group, ineffectiveness was a reason to drop out of the study.

Regarding HRQoL, the absolute QOLHEQ score was decreased (improvement in HE‐specific HRQoL) in both groups after 12 and 24 weeks compared to baseline. The mean percentage decrease in QOLHEQ score was 57.8% in the alitretinoin group versus 36.6% in the azathioprine group after 24 weeks (see Figure S1B) (*p* = 0.058). The minimal important change (MIC) of 22 points improvement was achieved by 64.3% of the patients in the alitretinoin group and by 14.3% of the patients in the azathioprine group at Week 24 (*p* = 0.063).

**FIGURE 1:**
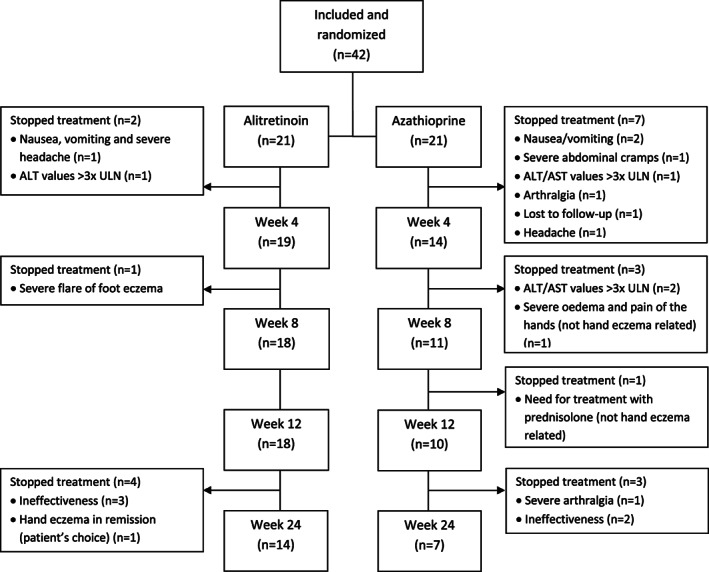
Flow chart of included patients. ULN, upper limit of normal

In both groups, patients mainly dropped out because of adverse events: three out of 21 patients (14.3%) in the alitretinoin group and nine out of 21 patients (42.9%) in the azathioprine group. An overview of all reasons for dropout can be found in the flowchart (see Figure 1). In the alitretinoin group, one patient (4.8%) discontinued treatment due to objective adverse events (alanine aminotransferase [ALT] or aspartate aminotransferase [AST] elevated >3× upper limit of normal) and two patients (9.5%) due to subjective adverse events (e.g., headaches). In the azathioprine group, this was respectively three patients (14.3%) due to objective adverse events (e.g., ALT or AST elevated >3× upper limit of normal) and seven patients (33.3%) due to subjective adverse events (e.g., nausea, abdominal cramps and arthralgia). During this trial, one serious adverse event occurred in a patient treated with azathioprine, consisting of viral gastroenteritis for which hospital admission was needed. During this patient's hospital admission, azathioprine was continued and the gastroenteritis was completely resolved.

## DISCUSSION

In this study, we observed a drop‐out rate of 57.1% due to adverse events and ineffectiveness in the azathioprine group. This number is significantly higher compared to a randomized controlled trial (RCT) in HE^6^ and an RCT in AD.^7^ However, dosage of azathioprine significantly differed from our study, which might explain the difference in dropout rates. The RCT in HE (no dropouts) used a low dose of 50 mg daily. This is currently the loading dose in the Dutch guidelines for the treatment of AD.^8^ The RCT in AD (13.6% dropout rate) used an initial dose of 1.5 mg/kg daily for all patients in that study, instead of 2.5 mg/kg daily, which the majority of patients (18/21) received in our study based on normal or high thiopurine S‐methyltransferase activity. The addition of allopurinol could lead to less drop‐outs due to ineffectiveness and adverse events, by shifting the metabolism of azathioprine to 6‐TGN production instead of 6‐MMP.^9^

The design of this study might have contributed to the high drop‐out rate. Since it was an open‐label head‐to‐head study: patients knew which treatment they received, including its side‐effects. This knowledge, giving patients certain expectations, plus the knowledge of other existing treatment options, for example, alitretinoin instead of azathioprine, might have made patients more eager to drop‐out of the study compared to blinded and placebo‐controlled studies.

In conclusion, both alitretinoin and azathioprine gave improvement of HE severity and HE‐specific HRQOL in this study. Alitretinoin might give more improvement of severity scores and HRQoL, as found in the analysis of our data, but we are not able to draw any conclusions on differences in efficacy between the two treatments due to the study being underpowered and the study results are biased by the high drop‐out rate.

## AUTHOR CONTRIBUTIONS

**Angelique N. Voorberg:** Conceptualization; investigation; writing – original draft; writing – review and editing; visualization; validation; methodology; formal analysis; project administration; data curation; resources. **Esmé Kamphuis:** Formal analysis; project administration; data curation; resources; investigation; visualization; validation. **Wietske A. Christoffers:** Data curation; resources; project administration; writing – review and editing; visualization; validation; investigation. **Geertruida L. E. Romeijn:** Resources; data curation; project administration; validation; visualization; investigation. **Jart A. F. Oosterhaven:** Conceptualization; investigation; funding acquisition; writing – original draft; methodology; validation; visualization; writing – review and editing; project administration; data curation; supervision; resources. **Marie L. A. Schuttelaar:** Supervision; resources; data curation; writing – review and editing; project administration; writing – original draft; methodology; validation; visualization; investigation; conceptualization; funding acquisition.

## CONFLICT OF INTEREST

Marie L. A. Schuttelaar is an advisor, consultant, speaker and/or investigator for AbbVie, Pfizer, LEO Pharma, Regeneron, Sanofi Genzyme, Eli Lilly and Galderma. She has received grants from Regeneron, Sanofi Genzyme, Novartis and Pfizer. Wietske A. Christoffers is a speaker for Sanofi Genzyme, and has been a consultant for LEO Pharma. The remaining authors declare no conflict of interest.

## ETHICS STATEMENT

This study was approved by the Dutch national competent authority (the Central Committee on Research Involving Human Subjects, reference number NL52232.042.15) and the local Ethical Review Board of the University Medical Center Groningen (METc 2015/176).

## Supporting information


**Appendix S1** Supporting information

**Appendix S2** Supporting information
