# Supplementary material for: Efficacy and safety of oral alitretinoin versus oral azathioprine in patients with severe chronic hand eczema: Results from a prematurely discontinued randomized controlled trial
Source: Contact Dermatitis. 2022 Jun 1;87(4):366–8. doi: 10.1111/cod.14161 (PMC9540441; doi:10.1111/cod.14161)
Supplement: Supplementary file 1 — Appendix S1 Supporting information [file COD-87-366-s002.pdf]

Appendix S1 - Supplemental Information

Efficacy and safety of oral alitretinoin versus oral azathioprine in patients with severe chronic hand eczema: results from a prematurely discontinued randomized controlled trial.

Angelique N. Voorberg, Esmé Kamphuis, Wietske A. Christoffers, Geertruida L.E. Romeijn, Jart A.F. Oosterhaven, Marie L.A. Schuttelaar

Supplemental Methods

Design, setting, and participants

This study was designed as a prospective randomized open-label study with blinded outcome assessment. It was conducted at the Department of Dermatology of the University Medical Center of Groningen (UMCG), the Netherlands, which is a tertiary referral center. The study population existed of adult patients, aged between 18 and 75 years old. Patients with subtypes other than palmar hyperkeratotic HE, were included. These subtypes of HE, e.g. chronic fissured HE and vesicular HE, were diagnosed following the criteria of the Danish Contact Dermatitis Group.[1] All patients needed to have a minimum severity of severe HE, which was graded using the validated photographic guide.[2] The full protocol, including all inclusion and exclusion criteria, can be found in **Appendix S2**. This study was approved by the Dutch national competent authority (the Central Committee on Research Involving Human Subjects, reference number NL52232.042.15) and the local Ethical Review Board of the University Medical Center Groningen (METc 2015/176).

Treatment

Eligible patients were randomized in a 1:1 ratio to 24 weeks of treatment with either alitretinoin 30mg daily or azathioprine 1.5 or 2.5mg/kg daily in two doses. Patients with a low or absent TPMT activity were excluded from the study, patients with intermediate TPMT activity received 1.5mg/kg daily and patients with normal to high TPMT activity received 2.5mg/kg daily.[3] Dose reduction during the study was permitted in both groups in case of abnormal findings from the physical examination or laboratory tests, or if adverse events occurred.

During the study, patients were allowed to continue using emollients and a topical corticosteroid (maximum class II) if necessary. In case of an exacerbation, patients were allowed to receive a maximum of three courses of rescue medication (mometasone furoate ointment once daily for one week). Women of childbearing potential were required to take monthly pregnancy tests and

to use proper contraception methods during treatment, and at least one month before and after treatment.

Outcomes

The primary endpoint for efficacy was response to treatment, defined as at least two steps improvement at week 24 on the photographic guide, a validated five point physician rated global assessment tool, covering five degrees of severity: clear, almost clear, moderate, severe and very severe.[2] To be able to make better comparisons with current randomized clinical trials for HE[4,5], achievement of clear/almost clear on the photographic guide at week 24 was assessed as an additional endpoint for efficacy.

Other secondary endpoints for efficacy were improvement in severity based on the hand eczema severity index (HECSI) and the patient global assessment (PaGA).[6] The HECSI is a physician rated severity assessment tool in which the severity of erythema, induration/papules, vesicles, scaling, fissures and scaling, and the percentage of the affected area are graded. The HECSI ranges from 0 to 360 points, with higher scores reflecting more severe disease.[6] The PaGA reflects the severity of hand eczema from the patient's perspective, covering six degrees of improvement: 'clear or almost clear' (at least 90% clearing of disease signs and symptoms compared to baseline), 'marked improvement' (at least 75% clearing), 'moderate improvement' (at least 50% clearing), 'mild improvement' (at least 25% clearing), 'no change', or 'worsening'.[7] For HE specific health related quality of life (HRQoL), the Quality of Life in Hand Eczema Questionnaire (QOLHEQ) was used.[8] This instrument contains 30 questions covering four subscales and ranges from 0 to 110[9], with higher scores reflecting worse quality of life, and the minimally important change (MIC) for improvement is 22 points. The photographic guide and HECSI were assessed during every visit, while the QOLHEQ was performed at baseline, week 12 and 24, and the PaGA was performed at week 12 and 24. All investigator rated severity outcomes were performed by a blinded trained nurse.

Furthermore, patients were asked about concomitant medication use and the occurrence of any adverse events during every study visit. Also, laboratory measurements were conducted corresponding to the summary of product characteristics (SmPC) safety profiles of both alitretinoin and azathioprine.[10,11]

Patient data including age, sex, body mass index, smoking, age of onset of HE and possible contributing etiological factors (e.g. atopic dermatitis (AD), irritant contact dermatitis, sensibilization to contact allergens) were collected at baseline.

Statistics

A prior study on the efficacy of alitretinoin in HE reported a responders ratio of 33% in HE subtypes other than hyperkeratotic HE[7], but literature on the efficacy of azathioprine on HE is lacking. Based on clinical experience, we estimated a responders ratio of 60%. With the anticipation of a maximal drop-out rate of 10%, we calculated that 58 patients in each treatment group would be necessary to reject the null hypothesis of no difference between alitretinoin and azathioprine, using a two-sided 0.05 significance level and with 80% power. Information on randomization, blinding, handling of missing items, and treatment allocation can be found in **Appendix S2**.

All continuous endpoints were analyzed using a mixed-effect model with repeated measures. HECSI and QOLHEQ values are presented as the mean percentage change with errors bars reflecting the standard deviation at the various time points compared to baseline. The Pearson $\chi 2$-test, or in case of violation of its assumptions the Fisher's exact test, was used to compare percentages in independent groups. Calculations were performed with IBM SPSS Statistics for Windows, Version 23.0 (IBM Corp., Armonk, New York). A P-value of <.05 was regarded as statistically significant.

Supplemental Tables

Table S1. Baseline characteristics of study population (*n*=42)

**Baseline characteristics**	**Alitretinoin group** (n=21)		**Azathioprine group** (n=21)	
Age, mean (SD)	48.8	(15.7)	52.8	(18.2)
Sex, *n* (%)				
• Male	6	(28.6)	11	(52.4)
• Female	15	(71.4)	10	(47.6)
BMI, median [IQR]	28.7	[24.9-33.4]	27.8	[24.6-33.0]
Smoking				
• Current smoker, *n* (%)	7	(33.3)	7	(33.3)
• Ex-smoker, *n* (%)	8	(38.1)	10	(47.6)
• Packyears, mean (SD)	13.9	(15.7)	20.4	(22.2)
Age of onset of HE, mean (SD)	35.9	(18.8)	42.7	(18.8)
Baseline photographic guide score, n (%)				
• Severe	14	(66.7)	17	(81.0)
• Very severe	7	(33.3)	4	(19.0)
Baseline HECSI score, median [IQR]	94.5	[65.5-121.5]	81.0	[69.0-108.0]
Baseline QOLHEQ score, median [IQR]	67.0	[58.5-84.0]	73.0	[46.5-87.0]
Morphological classification, n (%)				
• Chronic fissured	13	(61.9)	13	(61.9)
• Recurrent vesicular	8	(38.1)	8	(38.1)
Possible contributing etiological factors, n (%)				
• Irritant factors	18	(85.7)	15	(71.4)
• History of atopic dermatitis	7	(33.3)	6	(28.6)
• Current atopic dermatitis	3	(14.3)	0	(0.0)
• At least one positive reaction to the European baseline series, n	8	(38.1)	14	(66.7)
• Metals	3	(14.3)	6	(28.6)
• Preservatives	3	(14.3)	4	(19.0)
• Fragrances	3	(14.3)	1	(4.8)
• Rubbers	2	(9.5)	3	(14.3)
• Dyes/colors	3	(14.3)	1	(4.8)
• Topical medicaments	0	(0.0)	1	(4.8)
• Corticosteroids	1	(4.8)	2	(9.5)
• Other	1	(4.8)	4	(19.0)
TMPT activity, n (%)				
• Intermediate activity	-		3	(14.3)
• Normal/high activity	-		18	(85.7)

There were no significant differences between groups. *BMI*, body mass index; *HE*, hand eczema; *HECSI*, Hand Eczema Severity Index; *IQR*, interquartile range; *QOLHEQ*, Quality Of Life Hand Eczema Questionnaire; *SD*, standard deviation; *TPMT*, thiopurine methyltransferase.

Supplemental Figures

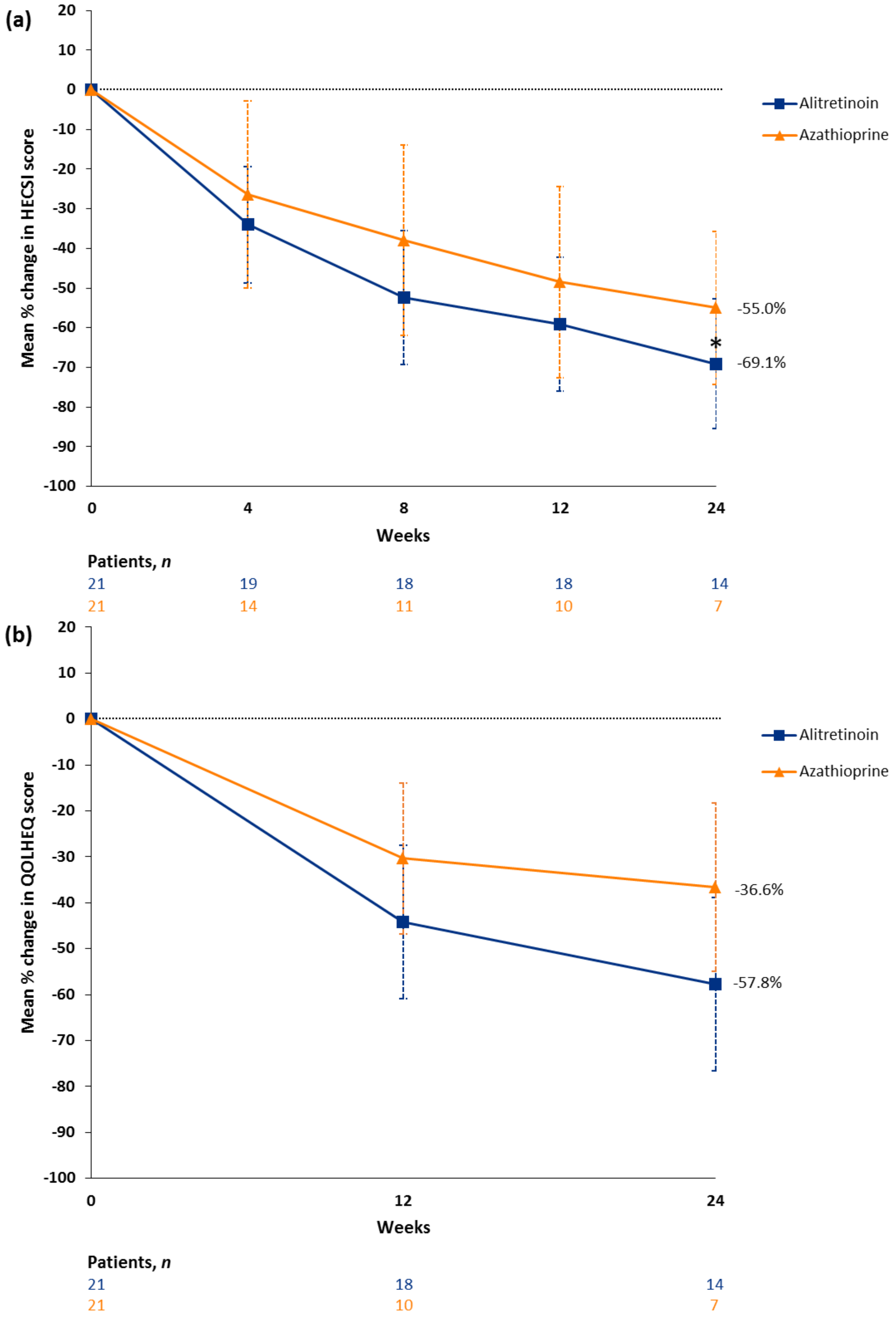

Figure S1. Hand Eczema Severity Index (HESCI) (a) and Quality of Life in Hand Eczema (QOLHEQ) score (b) development during 24 weeks of treatment with either alitretinoin or azathioprine. Negative values indicate improvement. The error bars reflect the 95% confidence intervals. *P<0.05.

Supplemental References

1. Menné T, Johansen JD, Sommerlund M, Veien NK. Hand eczema guidelines based on the Danish guidelines for the diagnosis and treatment of hand eczema. *Contact Dermatitis*. 2011;65(1):3-12.

2. Coenraads PJ, Van Der Walle H, Thestrup-Pedersen K, et al. Construction and validation of a photographic guide for assessing severity of chronic hand dermatitis. *Br J Dermatol*. 2005;152(2):296-301.

3. Meggitt SJ, Anstey AV, Mohd Mustapa MF, Reynolds NJ, Wakelin S. British Association of Dermatologists' guidelines for the safe and effective prescribing of azathioprine 2011. *Br J Dermatol*. 2011;165(4):711-734.

4. ClinicalTrials.gov. Identifier NCT04872101, Efficacy and Safety of Delgocitinib Cream in Adults With Moderate to Severe Chronic Hand Eczema (DELTA 2).

5. ClinicalTrials.gov. Identifier NCT03683719, Phase 2b Dose-ranging Trial to Evaluate Delgocitinib Cream 1, 3, 8, and 20 mg/g Compared to Delgocitinib Cream Vehicle Over a 16-week Treatment Period in Adult Subjects With Chronic Hand Eczema.

6. Held E, Skoet R, Johansen JD, Agner T. The hand eczema severity index (HECSI): a scoring system for clinical assessment of hand eczema. A study of inter- and intraobserver reliability. *Br J Dermatol*. 2005;152(2):302-307.

7. Ruzicka T, Lynde CW, Jemec GB, et al. Efficacy and safety of oral alitretinoin (9-cis retinoic acid) in patients with severe chronic hand eczema refractory to topical corticosteroids: Results of a randomized, double-blind, placebo-controlled, multicentre trial. *Br J Dermatol*. 2008;158(4):808-817.

8. Ofenloch RF, Weisshaar E, Dumke A-K, Molin S, Diepgen TL, Apfelbacher C. The Quality of Life in Hand Eczema Questionnaire (QOLHEQ): validation of the German version of a new disease-specific measure of quality of life for patients with hand eczema. *Br J Dermatol*. 2014;171(2):304-312.

9. Ofenloch RF, Oosterhaven JA, Susitaival P, et al. Cross-Cultural Validation of the Quality of Life in Hand Eczema Questionnaire (QOLHEQ). J Invest Dermatol. 2017;137(7):1454-1460.

10. Stiefel. Toctino (alitretinoin) 30 mg capsules SmPC. https://www.medicines.org.uk/emc/product/7543/smpc. Published 2021. Accessed December 24, 2021.

11. Aspen. Imuran (azathioprine) tablets 50mg SmPC. 2021. https://www.medicines.org.uk/emc/product/3823/smpc. Accessed December 24, 2021.
