# Supplementary material for: Efficacy and safety of oral alitretinoin versus oral azathioprine in patients with severe chronic hand eczema: Results from a prematurely discontinued randomized controlled trial
Source: Contact Dermatitis. 2022 Jun 1;87(4):366–8. doi: 10.1111/cod.14161 (PMC9540441; doi:10.1111/cod.14161)
Supplement: Supplementary file 2 — Appendix S2 Supporting information [file COD-87-366-s001.pdf]

Appendix S2

RESEARCH PROTOCOL

(August 2015)

PROTOCOL TITLE: Efficacy of oral alitretinoin versus oral azathioprine in patients with severe chronic non-hyperkeratotic hand eczema. A randomized prospective open-label trial with blinded outcome assessment.

**Protocol ID**	**52232**
**Short title**	**Alitretinoin vs azathioprine in severe chronic non-hyperkeratotic hand eczema**
**EudraCT number**	**2015-001447-37**
**Version**	**3.0**
**Date**	**18 August 2015**
**Coordinating investigator/project leader**	***Dr. M.L.A. Schuttelaar***<br>**University Medical Center Groningen**<br>**Dermatology department**<br>**Hanzeplein 1**<br>**9700 RB Groningen**<br>**050-361 2520**<br>**m.l.a.schuttelaar@umcg.nl**
**Principal investigator (in Dutch: hoofdonderzoeker/ uitvoerder)**	***Dr. M.L.A. Schuttelaar***<br>**University Medical Center Groningen**<br>**Dermatologie department**<br>**Hanzeplein 1**<br>**9700 RB Groningen**<br>**050-361 2520**<br>**m.l.a.schuttelaar@umcg.nl**
**Sponsor (in Dutch: verrichter/opdrachtgever)**	**University Medical Center Groningen**<br>**Dermatology department**<br>**Hanzeplein 1**<br>**9700 RB Groningen**
**Subsidising party**	***Not applicable***
**Independent physician**	***Dr. M.J. Wiegman***<br>**University Medical Center Groningen**<br>**Dermatology department**

	**050-3615689**<br>**m.j.wiegman@umcg.nl**
**Independent expert (advisor)**	***Prof. Dr. P.J. Coenraads***<br>**University Medical Center Groningen**<br>**Dermatology department**<br>**050-361 2869**<br>**p.j.coenraads@umcg.nl**
**Laboratory sites**	***Hospital laboratory University Medical Center Groningen (routine measurements)***
**Pharmacy**	***Not applicable (daily practice study)***

PROTOCOL SIGNATURE SHEET

Name	Signature	Date
**Head of Department:**<br>***Prof. dr. M.F. Jonkman***	[illegible]	21.8.15
**Principal Investigator:**<br>***Dr. M.L.A. Schuttelaar***	[illegible]	21-AUG-2015
**Investigator:**<br>***J.A.F. Oosterhaven, MSc***	[illegible]	21-AUG-2015

TABLE OF CONTENTS

1. INTRODUCTION AND RATIONALE 10
2. OBJECTIVES 12
3. STUDY DESIGN 13
4. STUDY POPULATION 14
   - 4.1 Population (base) 14
   - 4.2 Inclusion criteria 14
   - 4.3 Exclusion criteria 15
   - 4.4 Sample size calculation 16
5. TREATMENT OF SUBJECTS 18
   - 5.1 Investigational product/treatment 18
   - 5.2 Use of co-intervention 18
   - 5.3 Escape medication 18
6. INVESTIGATIONAL PRODUCT 19
   - 6.1 Name and description of investigational products 19
   - 6.2 Summary of findings from non-clinical studies 19
   - 6.3 Summary of findings from clinical studies 21
   - 6.4 Summary of known and potential risks and benefits 22
   - 6.5 Description and justification of route of administration and dosage 23
   - 6.6 Dosages, dosage modifications and method of administration 24
   - 6.7 Preparation and labelling of Investigational Medicinal Product 24
   - 6.8 Drug accountability 24
7. NON-INVESTIGATIONAL PRODUCT 25
   - 7.1 Name and description of non-investigational products 25
   - 7.2 Summary of findings from non-clinical studies 25
   - 7.3 Summary of findings from clinical studies 25
   - 7.4 Summary of known and potential risks and benefits 25
   - 7.5 Description and justification of route of administration and dosage 25
   - 7.6 Dosages, dosage modifications and method of administration 25
   - 7.7 Preparation and labelling of Non Investigational Medicinal Product 25
   - 7.8 Drug accountability 25
8. METHODS 26
   - 8.1 Study parameters/endpoints 26
     - 8.1.1 Main study parameter/endpoint 26
     - 8.1.2 Secondary study parameters/endpoints 26
     - 8.1.3 Other study parameters 28
   - 8.2 Randomisation, blinding and treatment allocation 28
   - 8.3 Study procedures 28
   - 8.4 Withdrawal of individual subjects 33
     - 8.4.1 Specific criteria for withdrawal 33
   - 8.5 Replacement of individual subjects after withdrawal 33
   - 8.6 Follow-up of subjects withdrawn from treatment 34

8.7 Premature termination of the study....34
9. SAFETY REPORTING....35
9.1 Section 10 WMO event....35
9.2 AEs, SAEs and SUSARs....35
9.2.1 Adverse events (AEs)....35
9.2.2 Serious adverse events (SAEs)....35
9.2.3 Suspected unexpected serious adverse reactions (SUSARs)....36
9.3 Annual safety report....37
9.4 Follow-up of adverse events....37
9.5 Data Safety Monitoring Board (DSMB)....37
10. STATISTICAL ANALYSIS....38
10.1 Primary study parameter....38
10.2 Secondary study parameters....38
10.3 Other study parameters....40
10.4 Interim analysis....40
Not applicable....40
11. ETHICAL CONSIDERATIONS....41
11.1 Regulation statement....41
11.2 Recruitment and consent....41
11.3 Objection by minors or incapacitated subjects....41
11.4 Benefits and risks assessment, group relatedness....41
11.5 Compensation for injury....42
11.6 Incentives....42
12. ADMINISTRATIVE ASPECTS, MONITORING AND PUBLICATION....43
12.1 Handling and storage of data and documents....43
12.2 Monitoring and Quality Assurance....43
12.3 Amendments....43
12.4 Annual progress report....44
12.5 End of study report....44
12.6 Public disclosure and publication policy....44
13. STRUCTURED RISK ANALYSIS....45
13.1 Potential issues of concern....45
13.2 Synthesis....49
14. REFERENCES....50

LIST OF ABBREVIATIONS AND RELEVANT DEFINITIONS

**6-MP**	**6-mercaptopurine**
**γ-GT**	**Gamma-Glutamyl Transferase**
**AD**	**Atopic Dermatitis**
**AE**	**Adverse Event**
**ALAT**	**Alanine aminotransferase**
**ALP**	**Alkaline phosphatase**
**ASAT**	**Aspartate aminotransferase**
**AUC**	**Area-Under-the-Curve**
**BCG**	**Bacillus Calmette-Guérin**
**CCMO**	**Central Committee on Research Involving Human Subjects; in Dutch: Centrale Commissie Mensgebonden Onderzoek**
**CI**	**Confidence Interval**
**CK**	**Creatine Kinase**
**CRF**	**Case Report Form**
**CV**	**Curriculum Vitae**
**DSMB**	**Data Safety Monitoring Board**
**EQ-5D-5L**	**EuroQol 5 dimensions 5 levels Health related Quality of Life questionnaire**
**HRQoL**	**Health Related Quality of Life**
**FTU**	**Finger-Tip-Unit**
**GCP**	**Good Clinical Practice**
**GMP**	**Good Manufacturing Practice**
**HECSI**	**Hand Eczema Severity Index**
**IC**	**Informed Consent**
**IGA**	**Investigator Global Assessment**
**LDL**	**Low Density Lipoprotein**
**METC**	**Medical research ethics committee (MREC); in Dutch: medisch ethische toetsing commissie (METC)**
**PaGA**	**Patient Global Assessment**
**PGA**	**Physician Global Assessment**
**PROM**	**Patient rated outcome measure**
**QOLHEQ**	**Quality of Life in Hand Eczema Questionnaire**
**RCT**	**Randomized Controlled Trial**
**(S)AE**	**(Serious) Adverse Event**

**SCORAD**	**Severity Scoring of Atopic Dermatitis**
**SPC**	**Summary of Product Characteristics (in Dutch: officiële productinfomatie IB1-tekst)**
**Sponsor**	**The sponsor is the party that commissions the organisation or performance of the research, in the case of this protocol embodied by the University Medical Center Groningen**
**SUSAR**	**Suspected Unexpected Serious Adverse Reaction**
**T4**	**Thyroxine**
**TGN**	**Thioguanine-nucleotide**
**TPMT**	**Thiopurine methyltransferase**
**TSH**	**Thyreoid Stimulating Hormone**
**UV**	**Ultraviolet**
**WMO**	**Medical Research Involving Human Subjects Act (in Dutch: Wet Medisch-wetenschappelijk Onderzoek met Mensen**

SUMMARY

Rationale: hand eczema is a common condition with a 1-year period prevalence up to 10%. Systemic treatment with alitretinoin is registered for all clinical types of hand eczema. However, it is especially effective in the hyperkeratotic subtype, and less effective in non-hyperkeratotic forms. Azathioprine is often prescribed for hand eczema in daily practice, and has a beneficial effect in non-hyperkeratotic subtypes. A few small studies support this observation. The efficacy of azathioprine in non-hyperkeratotic hand eczema could prove superior to that of alitretinoin.

Objective: to compare the efficacy of alitretinoin and azathioprine in the treatment of severe chronic non-hyperkeratotic hand eczema.

Study design: randomized prospective open-label trial with blinded outcome assessment, set in a university dermatology clinic, tertiary referral center. Assessment of severity and laboratory measurements in this study will be conducted corresponding to daily practice in our department.

Study population: adult patients (age 18-75 years) with severe chronic non-hyperkeratotic hand eczema, visiting the Department of Dermatology of the University Medical Center Groningen.

Intervention: group I: alitretinoin 30mg once daily. Group II: azathioprine 1.5 or 2.5mg/kg/day in 2 doses. The treatment period is 24 weeks.

Main study parameters/endpoints: the primary endpoint for efficacy is response to treatment, defined as an improvement of ≥ 2 steps on the Physician Global Assessment (PGA), developed by Ruzicka et al, after 24 weeks of treatment.

Secondary endpoints are improvement in: mean PGA at 12 and 24 weeks, the Hand Eczema Severity Index (HECSI) score, the Quality Of Life Hand Eczema Questionnaire (QOLHEQ), and a Patient Global Assessement (PaGA) of improvement. Adverse events will be registered, as well as time to response. Furthermore cost-utility, quality adjusted life years (QALYs) and cost-effectiveness will be assessed with the EQ-5D-5L questionnaire while monitoring treatment related costs.

Nature and extent of the burden and risks associated with participation, benefit and group relatedness: this trial is designed in a way that minimizes the burden and risks for the patient, because it will be carried out according to daily practice at our department. No additional laboratory measurements will be carried out next to measurements that are routinely assessed in treatment with one of both study drugs. One extra visit is needed to give patients consideration time before participation in the study. Results of the trial can be related to the population of patients with severe chronic non-hyperkeratotic hand eczema.

1. INTRODUCTION AND RATIONALE

Hand eczema is a common skin disease of the hands. It can have far-reaching personal, psychological and occupational consequences that may have a drastic impact on the life of those affected. A point prevalence of 4% and a 1-year-period prevalence up to 10% in the general population in Sweden have been reported. (1) A Danish study in young adults showed an incidence of 8.8 per 1000 person-years, a point prevalence of 7.1% and a 1-year-period prevalence of 14.3%. Women are significantly more often affected than men. (2)

The clinical presentation of hand eczema varies widely, ranging from erythematous fissured skin to a vesicular eruption or palmar hyperkeratosis. The disease could also be approached etiologically, considering exogenous factors causing contact allergy (e.g. nickel, perfumes) and irritant contact dermatitis (e.g. water, soap) in addition to endogenous factors like atopic dermatitis. (3)

There is general consensus concerning the first line treatment of hand eczema in various guidelines. Emollients and topical corticosteroids are considered to be the mainstay of treatment in mild forms. If these fail, secondary options like phototherapy and systemic treatment are available. (3-6) A recent Cochrane review (unpublished), however, states that conclusions on the best way to treat hand eczema cannot be drawn from the wide diversity of studies that were carried out up until now. More head-to-head trials are needed. (7)

Alitretinoin is the only registered systemic treatment option for all clinical types of severe chronic hand eczema in the Netherlands. It is currently the most investigated drug in terms of patient numbers in the second line treatment of severe chronic hand eczema. In well-designed, pharmaceutical sponsored trials, 30 mg alitretinoin a day resulted in a clear or almost clear response in 48% of the participants, compared to 17% in placebo. In the hyperkeratotic subtype 54% responded, compared to 12% in the placebo group. In two non-hyperkeratotic subgroups (defined as pompholyx and fingertip in the study) only 33% of participants reached clearance or almost clearance, compared to 12-30% in the placebo group. (8-10)

In the Netherlands, alitretinoin is registered since September 2013. It has never been compared to immunomodulating systemic drugs that are currently considered to be a second or third line alternative treatment for severe hand eczema. A trial comparing alitretinoin to cyclosporine ended prematurely due to the inability to include the total number of participants. (11)

In our clinical experience, supported by a retrospective assessment of patient data from our department (unpublished), azathioprine has beneficial effects on hand eczema. This concerns mainly the non-hyperkeratotic forms in which a response of 60% is estimated. A few small studies have shown that azathioprine may have a beneficial effect on hand

eczema. Scerri et al found a 75% good to excellent sustained clinical response in a case study in which pompholyx patients were treated with azathioprine. (12) In a case study by Pearce et al, 6 patients were treated with azathioprine for lymphoedema and hand dermatitis. 50% of patients reported a beneficial effect of the treatment. (13) In an Indian randomized comparative trial, conducted in 2013, chronic hand eczema patients were treated with either topical clobetasol propionate 0,05% cream alone, or in combination with azathioprine. A 91% improvement of hand eczema severity index (HECSI) score at 24 weeks was found in the azathioprine group, compared to 39% in the control group. (14)

In patients with atopic dermatitis (AD) who were treated with azathioprine a mean relative reduction in severity scoring of atopic dermatitis (SCORAD) was found in 39% of patients. Furthermore, an improvement to cleared, minimal or mild disease (investigator global assessment (IGA) <2) in 68.2% of all patients was found at 12 weeks. (15) Patients with AD frequently have involvement of the hands. (6) It is our clinical experience that these patients, when treated with systemic therapy, often show rapid healing of their hands as well.

Although these studies show favorable results, more studies on the use of azathioprine in the treatment of hand eczema are needed. This trial aims to compare alitretinoine to azathioprine in the treatment of severe chronic non-hyperkeratotic hand eczema. The study assesses the efficacy of both treatments and will show head-to-head results, which should contribute to uncovering the best treatment strategy for hand eczema.

2. OBJECTIVES

Primary objective: to compare the efficacy of alitretinoine and azathioprine in the treatment of patients with severe chronic non-hyperkeratotic hand eczema.

Secondary objectives:

- to compare time to response
- to compare health related quality of life
- to compare improvement in severity of hand eczema, assessed by the patient
- to compare safety
- to compare cost-utility and cost-effectiveness

3. STUDY DESIGN

This study is designed as a randomized prospective open label study (or RCT). It will be conducted at the Department of Dermatology of the University Medical Center of Groningen, a tertiary referral center. Assessment of disease severity, laboratory measurements and quality of life in this study will be conducted comparable to daily practice assessments at our department.

The duration of the study for an individual patient is 24 weeks. The expected total duration of the study is 1.5 years.

Flow chart of study design:

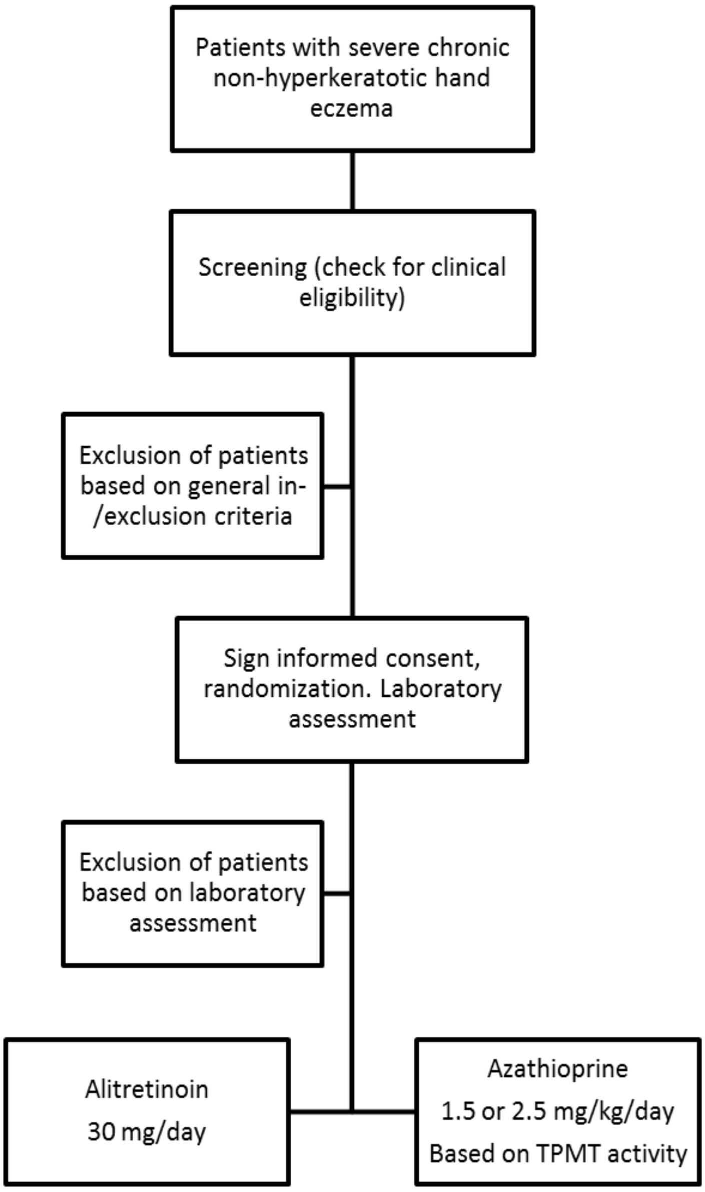

4. STUDY POPULATION

4.1 Population (base)

The study population will exist of adult patients with severe chronic non-hyperkeratotic hand eczema, visiting the Department of Dermatology of the University Medical Center Groningen.
Non-hyperkeratotic hand eczema will be diagnosed following the criteria of the Danish Contact Dermatitis Group. (6) The severity of the hand eczema at the screening will be graded by means of a Physician Global Assessment using a validated Photoguide. (16)

Woman in the fertile age will be required to use proper contraception methods. Men and women of all ethnicities of 18 years and older will be recruited.

Patients meeting all inclusion criteria, while not meeting any of the exclusion criteria, will be asked to participate.

Our sample size calculation showed a total of 116 patients need to be included. Given the fact that 300-350 individual patients with hand eczema visit our outpatient eczema clinic every year, and the mean severity is high because our department is a tertiary referral center, we believe this number to be feasible.

4.2 Inclusion criteria

In order to be eligible to participate in this study, a subject must meet all of the following criteria:

- Age ≥ 18 years and ≤ 75 years
- Severe chronic non-hyperkeratotic hand eczema for a minimum duration of 3 months as defined by a Physician Global Assessment (PGA) using a validated Photoguide (16)
- Refractory to standard therapy, defined as:
    - Patients received treatment with topical corticosteroids of class II or higher for at least 8 weeks within 3 months before enrolment, with either no response or a transient response
    - Patients had also received standard skin care, including emollients and barrier protection as appropriate, without significant improvement
    - Patients had avoided irritants and allergens, if identified, without significant improvement

- Women of childbearing potential are required to use at least two forms of contraception for at least 1 month before starting treatment, during treatment, and for at least 1 month after finishing treatment; these women are required to take monthly pregnancy tests
- Able to provide written Informed Consent
- Able to speak and read the Dutch language

4.3 Exclusion criteria

A potential subject who meets any of the following criteria will be excluded from participation in this study:

General criteria prior to randomization

- Treatment with alitretinoin or azathioprine in the previous 3 months
- Hyperkeratotic palmar eczema as defined by the Danish Contact Dermatitis Group (6)
- Patients with predominantly atopic dermatitis, in which the hands are also involved. Patients with mild atopic dermatitis, in which the hands are mainly affected are eligible for inclusion.
- Psoriasis
- Active bacterial, fungal, or viral infection of the hands
- Pregnant/lactating or planning to become pregnant during the study period
- Treatment with systemic medication or UV radiation within the previous 4 weeks
- Mentally incompetent
- Immunocompromised status
- Known or suspected allergy to ingredients in the study medications
- Inclusion in a study of an investigational drug within 60 days prior to start of treatment
- Current malignancy (other than successfully treated non-metastatic cutaneous squamous cell or basal cell carcinoma and⁄or localized carcinoma in situ of the cervix)
- Current active pancreatitis
- Living vaccine (including bacillus Calmette-Guérin (BCG), varicella, measles, mumps, rubella, yellow fever, oral polio and oral typhoid) in the last 2 weeks or the planned application of such a vaccine during the study period
- Evidence of alcohol abuse or drug addiction
- Chronic or recurrent infectious diseases
- Contact sensitizations with clinical relevance to the hands, in which exposure to allergens is not avoided
- Hypervitaminosis A due to the use of vitamin A supplements containing >2000 IU

- Use of drugs with potential to change the effective dosis of study drugs within the previous 2 weeks (see paragraph 5.2)

Laboratory exclusion criteria post randomization

- Alanine aminotransferase (ALAT) and⁄or aspartate aminotransferase (ASAT) values > 200% of the upper limit of normal
- Impaired renal function as indicated by a clinically relevant abnormal creatinine value (to be determined by investigator or treating physician)
- Anemia as indicated by a clinically relevant lowered hemoglobin value (to be determined by investigator or treating physician)

Alitretinoin specific

- Triglycerides > 200% of the upper limit of normal,
- Cholesterol or low density lipoprotein (LDL) cholesterol values > 200% of the upper limit of normal
- Uncontrolled hypothyroidism (to be determined by investigator or treating physician)

Azathioprine specific

- Patients with low or absent thiopurine methyltransferase (TPMT) activity (defined in our center as <52 nmol/gHb/hour, combined with genotyping showing homozygous of compound heterozygous mutations) and a subsequent risk for life-threatening myelotoxicity

4.4 Sample size calculation

This trial hypothesizes a superior response to azathioprine compared to alitretinoin in the treatment of severe chronic non-hyperkeratotic hand eczema.

A sample size of 52 in each group will have 80% power to be able to reject the null hypothesis of no difference between alitretinoin and azathioprine, using a chi-square test with a two-sided 0.05 significance level. In this calculation we used the following assumptions: randomization ratio is 1:1, and we expected the percentage of responders in the alitretinoin group to be 33% (10). Literature on the efficacy of azathioprine on hand eczema in terms of PGA is lacking. From a retrospective assessment of available patient data from our department, combined with clinical experience, we estimate 60% responders in the azathioprine group. We anticipate a drop-out of maximal 10% of randomized patients; a small percentage prior to first application of study drugs due to excluding laboratory measurements and a larger percentage during follow up, mainly due to subjective side effects. We therefore plan to include 116 patients in total, 58 in the alitretinoin group and 58 in the azathioprine group. (Calculated with the sample size

calculator of the Department of Biostatistics, Vanderbilt University, United States, available at http://biostat.mc.vanderbilt.edu/wiki/Main/PowerSampleSize.)

5. TREATMENT OF SUBJECTS

5.1 Investigational product/treatment

Group I will receive alitretinoin.

Group II will receive azathioprine.

Dosage, dosage modification and method of administration can be found in paragraph 6.6.

5.2 Use of co-intervention

All patients will be given an emollient cream with instructions to apply it frequently. One week before the first intake of study drugs, concomitant treatment with a topical class II corticosteroid at maximum is permitted when needed, with a maximum application of one finger-tip-unit (FTU) for each hand daily. (17) This also applies for concomitant topical corticosteroid therapy during the study period. Higher class topical corticosteroids are not allowed as maintenance therapy.

Generally prohibited concomitant treatments during therapy comprise: systemic corticosteroids, other retinoids, any other systemic or topical anti-eczema therapy, phototherapy, immunosuppressive or cytostatic drugs.

Alitretinoin specific prohibited concomitant treatment: vitamin A supplements, tetracyclines and azole antimycotics. St John’s wort should not be taken because of a possible interaction with hormonal anti-conceptive drugs. This could possibly result in a pregnancy, which is absolutely contraindicated because of the teratogenic nature of alitretion. (10)

Azathioprine specific prohibited concomitant treatment: allopurinol, febuxostat, ribavirin. Patients on cumarines are requested to inform their thrombosis service of treatment with azathioprine. Careful monitoring of coagulation is advised. (18)

5.3 Escape medication

In case of an exacerbation or postponed treatment effect, patients are allowed to receive a maximum of 3 courses of rescue medication: mometasone furoate once daily for 1 week, with a maximum application of one FTU for each hand daily. (17)

6. INVESTIGATIONAL PRODUCT

The products of this study will be used as in usual clinical practice.

6.1 Name and description of investigational products

Alitretinoin is a retinoid drug. The pharmacological action of retinoid drugs may be explained by their effects on cell proliferation, cell differentiation, apoptosis, angiogenesis, keratinization, sebum secretion and immunomodulation. Unlike other retinoids, which are specific agonists of either RAR or RXR receptors, alitretinoin binds to members of both receptor families. The mechanism of action of alitretinoin in chronic hand eczema is unknown. It has been shown that alitretinoin has immunomodulatory and anti-inflammatory effects that are relevant to skin inflammation. CXCR3 ligands and CCL20 chemokines, that are brought to expression in eczematous skin lesions, are down-regulated by alitretinoin in cytokine stimulated keratinocytes and dermal endothelium cells. In addition, alitretinoin suppresses the expansion of cytokine activated leukocyte subsets and antigen presenting cells. It has been observed that in humans alitretinoin only minimally affects sebum secretion.(10)

Azathioprine is a prodrug of 6-mercaptopurine (6-MP). 6-MP is inactive in itself, but works as a purine antagonist. It requires uptake into the cell and intracellular anabolism to thioguanine-nucleotides (TGN's) for immunosuppression. The TGN's and other metabolites (e.g. 6-methylmercaptopurine nucleotides) inhibit *de novo* purinesynthesis and interconversions of purine nucleotides. The TGN's are also incorporated in nucleic acids and this contributes to the immunosuppressive action of the drug. Another potential mechanism of action for azathioprine comprises the inhibition of multiple pathways in nucleic acid biosynthesis. This impedes the initiation and accumulation of an immune response. Due to the nature of these mechanisms, azathioprine takes several weeks to months to show its therapeutic effect. (18)

6.2 Summary of findings from non-clinical studies

Alitretinoin (from SPC text) (10)

Acute toxicity

As with other retinoids, the acute toxicity of alitretinoin was low in mice and rats. The LD50 after intraperitoneal administration was >4000 mg/kg after 24 hours and 1400 mg/kg after 10 days. The approximate LD50 after oral administration in rats was 3000 mg/kg.

Chronic toxicity

Alitretinoin was tested in long-term studies up to 9 months in dogs and 6 months in rats. Signs of toxicity were dose-related and occurred at exposures similar to the human therapeutic exposure based on area-under-the-curve (AUC). Effects were characteristic for retinoids (consistent with hypervitaminosis A), and were generally spontaneously reversible.

Teratogenicity

Like other retinoids, alitretinoin has been shown to be teratogenic in vitro and in vivo. Due to the teratogenic potential of alitretinoin, women of childbearing potential must adhere to strict pregnancy prevention measurers during and 1 month following alitretinoin therapy.

Fertility

Alitretinoin was tested in a study of fertility and early embryonic development in rats. No effects on male or female reproductive parameters were observed at the highest dose tested. However, systemic exposure in this study did not reach the level observed in patients.

As with other retinoids reversible effects on male reproductive organs were observed in experimental animals in the form of disturbed spermatogenesis and associated degenerative lesions of the testes. The safety margin in dogs with regard to the no-effect level of toxicity to male reproductive organs was 1-6 for a human dose of 30 mg.

Mutagenicity

In in vitro or in vivo tests, alitretinoin has been shown not to be mutagenic.

Carcinogenicity

Alitretinoin was tested in 2-year carcinogenicity studies in rats and mice. Dose-related retinoid-specific toxicity was seen at higher doses, but no carcinogenic potential was noted.

Phototoxicity

Alitretinoin was found to be phototoxic in vitro and in vivo

Azathioprine (from SPC text) (18)

Toxicological studies in animals have shown that the hematopoietic system is most strongly influenced by depression of mainly granulopoiesis and relative sparing of megakaryocytes and thus the formation of thrombocytes. In dogs azathioprine caused

death due to agranulocytosis. Related to the effect on hematopoiesis is the effect on the lymphatic system, with atrophy of the lymphatic tissue as was found in rhesus monkeys. Azathioprine, like 6-MP, can damage the liver. In dogs a reversible hepatotoxicity was noted. Dogs are very sensitive to this reaction.

Teratogenicity or embryolethality has been seen in a number of animal species with varying degree of susceptibility. In rabbits, a dose of 5-15 mg/kg body weight daily on days 6-14 of pregnancy produced skeletal abnormalities, in mice and rats, doses of 1-2 mg/kg body weight daily on days 3-12 were lethal to embryos.

Evidence of teratogenicity of azathioprine in man is equivocal. As with all cytotoxic chemotherapy, contraceptive precautions should be advised when either partner is receiving azathioprine.
Azathioprine was mutagenic in a number of in-vitro and in-vivo genotoxicity assays.

In long-term carcinogenicity studies of azathioprine in mice and rats, an increased incidence of lymphosarcomas (mice) and epithelial tumours and carcinomas (rats) were observed at dosages that were up to 2-fold the human therapeutic dosage.

6.3 Summary of findings from clinical studies

Alitretinoin

In well-designed, pharmaceutical sponsored trials, 30 mg alitretinoin a day resulted in a clear or almost clear response in 48% of the participants, compared to 17% in placebo. In the hyperkeratotic subtype 54% responded, compared to 12% in the placebo group. In two non-hyperkeratotic subgroups (defined as pompholyx and fingertip in the study) only 33% of participants reached clearance or almost clearance, compared to 12-30% in the placebo group. (8-10) Retreatment of patients that previously had a response to alitretinoin treatment led to a second response to a new course of treatment in 80% of patients. Retreatment was well tolerated. (19)

Azathioprine

A few small studies have shown that azathioprine may have a beneficial effect on hand eczema. Scerri et al found a 75% good to excellent sustained clinical response in a case study in which pompholyx patients were treated with azathioprine. (12) In a case study by Pearce et al, 6 patients were treated with azathioprine for lymphoedema and hand dermatitis. 50% of patients reported a beneficial effect of the treatment. (13) In an Indian randomized comparative trial, conducted in 2013, chronic hand eczema patients were

treated with either topical clobetasol propionate 0,05% cream alone, or in combination with azathioprine. A 91% improvement of hand eczema severity index (HECSI) score at 24 weeks was found in the azathioprine group, compared to 39% in the control group. (14)

6.4 Summary of known and potential risks and benefits

The intended benefit of study drugs is to reduce the severity of hand eczema.

Main risks in the *alitretinoin* group are (10):

- Teratogenicity of the study drugs
- Occurrence of allergic / anaphylactic reactions
- Depression with anxiety, mood changes and suicidal tendencies
- Sunburn
- Xerostomia, xerosis cutis
- Keratoconjunctivitis sicca, keratitis, blurred (night) vision, cataract. Care must be taken when driving a vehicle or when operating machines
- Myalgia, artralgia, increase of CK values
- Exostosis, ankylosing spondylitis
- Headache
- Blushing
- Increase of cholesterol and triglycerides, with ultimately pancreatitis
- Decrease of TSH and T4
- Increased liver transaminases
- Decrease in effective dose of simvastatin
- Change at scarring or dermatitis during therapy and 6 months after in case of aggressive dermabrasion or epilation
- Anemia
- Epistaxis
- Alopecia
- Benign intracranial hypertension (rare and most seen in combination with tetracyclines)
- Inflammatory bowel disease (rare)
- Vasculitis (rare)

Main risks in the *azathioprine* group are (18):

- Occurrence of allergic / anaphylactic reactions
- (Severe) myelumsupression (this can occur independent of TPMT activity)
- Hepatotoxicity

- Nausea
- Exacerbation of a varicella zoster virus infection
- Inhibition of anticoagulation effect of coumarines
- Possibly teratogenic
- Increased sensitivity to infections (viral, fungal, bacterial)
- Alopecia (rare)
- Progressive multifocal leucoencephalopathy (rare)
- Development of non-Hodgkin lymphoma, non-melanoma skin cancer, sarcoma and cervix carcinoma, related to duration of treatment with immunosuppressant (rare)
- Stevens-Johnson, toxic epidermal necrosis (rare)
- Reversible pneumonitis (rare)

6.5 Description and justification of route of administration and dosage

Group I will receive an oral *alitretinoin* capsule of 30mg once daily for a total of 24 weeks. In a dose-finding study, the effectiveness and tolerability of this dose was established and it is recommended to use this as the standard dose for the prescription of alitretinoin in hand eczema. (8,10)

Group II will receive oral *azathioprine* tablets twice daily in a dose of 1.5 or 2.5mg/kg/day, depending on the thiopurine methyltransferase (TPMT) activity. A blood sample to measure TPMT activity will be obtained at the randomization visit. In thiopurine metabolism, the activity of the gene that encodes for the expression of the TPMT enzyme has an important effect on the toxicity of thiopurines in the human body. Genetic interpersonal differences lead to a difference in TPMT activity. Patients with low or absent activity of TPMT (0.3% of patients) have a risk of fatal myelosuppression when given normal doses of thiopurines. The result of the test is available in 3-4 weeks in our center. Patients will be allowed to continue their current topical treatment (if this is in accordance with the in-/exclusion criteria) for three more weeks and are required to taper the strength of topical corticosteroids to maximum class 2 during the week prior to baseline.
At the azathioprine baseline visit (4 weeks following randomization visit) patients receive a dosage of azathioprine tailored to their TPMT activity. Patient with a low or absent TPMT activity (indicating homozygous or compound heterozygous mutations) will be excluded from the study. Patients with intermediate activity of TPMT (indicating heterozygous mutation) will receive 1.5mg/kg/day. Dosage in patients with normal to high activity levels of TPMT (indicating homozygous wild-type) will receive 2.5mg/kg/day. This is in accordance with current guidelines. (20)

6.6 Dosages, dosage modifications and method of administration

The study drugs will be distributed to participants in amounts that allow drug intake as per protocol up to the next visit, 4 weeks later.

Dosage reduction is allowed in both groups in case of abnormal findings on physical examination, laboratory markers, and/or adverse events. Alitretinoin dose can be reduced from 30mg/day to 10mg/day, in accordance with the SPC-text.

Alitretinoin capsules need to be administered orally once daily, during a meal.

Azathioprine tablets need to be administered orally with a glass of liquid (200ml), during a meal.

6.7 Preparation and labelling of Investigational Medicinal Product

Preparation and labelling of the study drugs will be carried out according to usual practice by the community pharmacy, honouring relevant GMP guidelines.

6.8 Drug accountability

Not applicable.

7. NON-INVESTIGATIONAL PRODUCT

Not applicable.

7.1 Name and description of non-investigational products

7.2 Summary of findings from non-clinical studies

7.3 Summary of findings from clinical studies

7.4 Summary of known and potential risks and benefits

7.5 Description and justification of route of administration and dosage

7.6 Dosages, dosage modifications and method of administration

7.7 Preparation and labelling of Non Investigational Medicinal Product

7.8 Drug accountability

8. METHODS

8.1 Study parameters/endpoints

8.1.1 Main study parameter/endpoint

Severity of hand eczema

The Physician Global Assessment (PGA) developed by Ruzicka et al, covers 5 degrees of severity (clear, almost clear, mild, moderate, severe) and takes into account the intensity and percentage of hand surface involved. (8,9) Response to treatment is defined as an improvement of ≥ 2 steps on the PGA. Severe hand eczema is defined as responding to treatment if a status of at least ‘mild’ is achieved. Moderate hand eczema is defined as responding to treatment if a status of at least ‘almost clear’ is achieved.

In this study the main endpoint is the between-group difference in response to treatment between baseline and 24 weeks of treatment.

8.1.2 Secondary study parameters/endpoints

Severity of hand eczema

- Between-group difference in mean change in PGA between baseline and 12 and 24 weeks.
- Between-group difference in response to treatment between baseline and 12 weeks of treatment.
- Between-group difference in mean change between baseline and week 4, 8, 12 and 24, assessed by the Hand Eczema Severity Index (HECSI) score. (21) The HECSI is an objective severity assessment based on clinical symptoms only. It includes erythema, fissures, vesicles, scaling, oedema, papules and measurement of the affected area. The score ranges from 0-360, with 360 indicating the most severe eczema.
- Between-group difference in time to response (time to first PGA improvement of ≥ 2 steps). This is only measured at control visits so possible outcome is limited to 4, 8, 12 and 24 weeks.

Patient rated outcome measures (PROMs)

Quality of life

- Between-group mean change in quality of life between baseline and 12 and 24 weeks, assessed by the Quality Of Life in Hand Eczema Questionnaire (QOLHEQ). The QOLHEQ is a multi domain disease specific instrument for

hand eczema assessing impairments in quality of life. The score ranges from 0-120, with 120 indicating worst quality of life.

Patient reported improvement

- Between-group difference in patients reporting improvement as ‘clear or almost clear’ at week 12 and 24, assessed by Patient Global Assessment (PaGA). The PaGA takes signs and symptoms into account. It covers 6 degrees of improvement: ‘clear or almost clear’ (at least 90% clearing of disease signs and symptoms compared to baseline), ‘marked improvement’ (at least 75% clearing), ‘moderate improvement’ (at least 50% clearing), ‘mild improvement’ (at least 25% clearing), ‘no change’, or ‘worsening’. (9)

Safety and tolerability

- Adverse events in both groups will be registered.

Cost-utility and cost-effectiveness

- Between-group difference in mean Quality Adjusted Life Years (QALY’s) will be measured by the EQ-5D-5L score at baseline, week 12 and week 24. The EQ-5D-5L is a measure for HRQoL and utility values. The EQ-5D-5L questionnaire includes a descriptive system, which comprises 5 dimensions of health: mobility, self-care, usual activities, pain/discomfort, and anxiety/depression. Moreover it includes a visual analog scale (VAS), which records the respondent's self-rated health status on a graduated (0–100) scale.
- Direct medical costs will be determined using standardized prices for consultation, treatment (medication; alitretinoin or azathioprine, topical treatment of corticosteroids and emollientia; if necessary oral or topical treatment of antibiotics), diagnostic tests, laboratory measurements, visits to the general practitioner for hand eczema and hospital admissions (in patient and/or daycare). Included patients will be asked to keep track of how much they spend on over-the-counter medication and other products for their hand eczema (out-of-pocket costs). Direct non-medical costs, consisting of travel costs, will be determined using average travel costs to the hospital as determined by relevant Dutch guidelines on cost-studies in healthcare. (22)
- Indirect costs, consisting mainly of productivity loss, will be also be calculated using tables from the guidelines with average income of Dutch workers stratified by age and gender, corrected for shift working / irregular working hours.

8.1.3 Other study parameters

At baseline age, sex, Body-Mass-Index, current and history of atopic dermatitis (both defined by U.K. Working Party criteria) (23), age of onset of atopic dermatitis, age of onset of hand eczema, current use of statins, current use of thyreomimetics, currently smoking and amount of pack-years will be registered because these might be confounders. (2,24) Pack years are calculated by multiplying the total years smoked with the average packs per day smoked over these years. (25) For this, we use the online Smoking Pack Years Calculator, created by dr NJ Masters and C Tutt. (26)

8.2 Randomisation, blinding and treatment allocation

Randomization is carried out by a computer program.
This is a study with blinded efficacy assessors, which are unaware of treatment allocation. The participants and treating physician will be aware of treatment allocation. Efficacy assessment will be carried out by physicians or specialized eczema nurses who are experienced in assessing hand eczema by PGA and HECSI in daily pratice. Blinding will only be broken after analyzing the data.
At screening visit (-1 week for *alitretinoin*, -5 weeks for *azathioprine*), clinical eligibility of patients is assessed. If eligible, patients are given a least 1 week time to consider participation in the study. If patients choose to participate they will be randomized to alitretinoin or azathioprine treatment (at week 0 for *alitretinoin*, -4 weeks for *azathioprine*). Based on the assigned group, appropriate laboratory assessments will be performed. If these assessments do not show abnormalities as described in the *laboratory exclusion criteria post randomization*, patients will receive the treatment.

8.3 Study procedures

<u>Procedures part of standard medical treatment:</u>
According to daily practice, a detailed patient history is obtained of all newly referred patients with hand eczema, and they are planned for patch testing to exclude contact allergy. During this first period, patients are treated with topical corticosteroids and emollients. Standard skin care advice is provided and exposure to provoking factors is discussed. If a relevant contact allergy is ruled out and the hand eczema proves to be refractory to topical therapy and/or UV therapy, the next step is systemic therapy. These patients are a candidate for the current study.
Laboratory analysis is performed to verify contra-indications for systemic treatment; in the current study alitretinoin or azathioprine. For azathioprine this check takes 4 weeks, due to the necessary measurement of TPMT (see paragraph 6.5). During therapy, standard

monitoring of blood values is carried out, according to SPC texts and current guidelines. At every visit, the Physician Global Assessment (PGA) for severity is determined (9) and the hand eczema is scored using the Hand Eczema Severity Index (HECSI, see appendix) (21), corresponding to our daily practice. Additionally, a Patient Global Assessment (PaGA) for improvement is obtained at week 12 and 24. Furthermore, health related quality of life is scored with a Dutch version of the Quality of Life in Hand Eczema Questionnaire (QOLHEQ, see appendix) at the start of therapy, at week 12 and week 24. (27)

Standard laboratory tests to be performed include:
Alitretinoin: at week 0, 4, 8, 12 and 24, laboratory tests are carried out, including: full blood count, ASAT, ALAT, ALP, γ-GT, serum creatinine, cholesterol, triglycerides, HDL, TSH, T4, glucose. At baseline, a pregnancy test will be carried out.
Azathioprine: at pre-baseline (week -4) TPMT activity will be measured, as described in paragraph 6.5. Furthermore, other laboratory tests are carried out, including: full blood count, ASAT, ALAT, ALP, γ-GT, bilirubine and serum creatinine. At week 1, 2, 4, 8, 12 and 24, these will be repeated.

<u>Procedures extra for this study:</u>
Patients will be given one week to consider participation. Due to this, one extra visit is needed to randomize the patient and obtain baseline data. A Patient Global Assessment (PaGA) one-item questionnaire will be obtained at week 12 and 24. This procedure is only extra in terms of obtaining a quantitative assessment of the qualitative report that a patient provides us in daily practice.

Patients will be asked to keep track of out-of-pocket costs on products for their hand eczema. During each visit, patients will be asked for direct and indirect medical and non-medical costs (see paragraph 8.1.2).

No diagnostic procedures or other treatments will be postponed for patients participating in this study. However, patients receive at least 1 week time to consider participation in the study.

<u>Study overview:</u>
In table 1 and 2 a systematic overview of the study for respectively the alitretinoin and azathioprine arm is presented.

Table 1. Study schedule – *alitretinoin* arm

Visit	V-1 Screening	V0 Baseline Ali	V1	V2	V3	V4
**Week**	**-1**	**0**	**4**	**8**	**12**	**24**
**Screening/baseline**						
**Check for clinical eligibility (inclusion/exclusion)**	x					
**Sign informed consent**		x				
**Randomization**		x				
**Baseline data / demographics / medical history / baseline costs**		x				
**Laboratory exclusion criteria post randomization**		x				
**Start medication**		x				
**Treatment**						
**Escape medication assessment**			x	x	x	x
**If applicable: dosage reduction assessment**			x	x	x	x
**Efficacy**						
**Severity scoring PGA / HECSI**		x	x	x	x	x
**Quality of life questionnaire QOLHEQ**		x			x	x

**PaGA**					x	x
**Costs**						
**Cost assessment**		x	x	x	x	x
**Cost-utility questionnaire EQ-5D-5L**		x			x	x
**Safety**						
**Lab control**		x	x	x	x	x
**Concomitant medication**		x	x	x	x	x
**Adverse events**			x	x	x	x
**If applicable: premature withdrawal assessment**		x	x	x	x	x

Table 2. Study schedule – *azathioprine* arm

Visit	V-1<br>Screening	V0<br>Baseline Aza	V0a<br>Additional<br>Baseline Aza	L<br>A<br>B	L<br>A<br>B	V1	V2	V3	V4
**Week**	**-5**	**-4**	**0**	**1**	**2**	**4**	**8**	**12**	**24**
**Screening/baseline**									
**Check for clinical eligibility (inclusion/exclusion)**	x								
**Sign informed consent**		x							
**Randomization**		x							
**Baseline data/ demographics / medical history / baseline costs**		x							
**TPMT assessment**		x							
**Laboratory exclusion criteria post randomization**			x						
**Dosage azathioprine baseline**			x						
**Start medication**			x						
**Treatment**									
**Escape medication assessment**						x	x	x	x
**If applicable: dosage reduction assessment**						x	x	x	x
**Efficacy**									
**Severity scoring PGA / HECSI**			x			x	x	x	x
**Quality of life questionnaire QOLHEQ**			x					x	x
**PaGA**								x	x
**Costs**									
**Cost assessment**		x	x			x	x	x	x
**Cost-utility questionnaire EQ-5D-5L**			x					x	x
**Safety**									
**Lab control**		x		x	x	x	x	x	x
**Concomitant medication**		x	x			x	x	x	x
**Adverse events**			x			x	x	x	x
**If applicable: premature withdrawal assessment**		x	x			x	x	x	x

Patients are permitted to deviate from the schedule with a maximum of 7 days during week 0-8. From week 9 a maximum deviation of 14 days is permitted.

8.4 Withdrawal of individual subjects

Subjects can leave the study at any time for any reason if they wish to do so without any consequences. The investigator can decide to withdraw a subject from the study for medical reasons.

8.4.1 Specific criteria for withdrawal

- Evidence of pregnancy
- Occurrence of serious adverse events
- Lack of efficacy at 12 weeks, defined as no improvement assessed by the PGA (at least 1 step improvement is necessary to continue treatment after 12 weeks)
- Use of prohibited concomitant therapy, or a need for their use
- The need for more than 3 courses of rescue medication
- Anaphylactic reaction or other severe systemic reaction to study drug intake
- Diagnosis of malignancy during study, excluding non-metastatic cutaneous squamous cell or basal cell carcinoma and/or localized carcinoma in situ of the cervix
- Any infection that is opportunistic and other infections whose nature or course may suggest an immunocompromised status
- Administration of a living vaccine
- Severe laboratory abnormalities including:
    - ALAT and/or ASAT values > 300% of the upper limit of normal
    - Lowered full blood count indicating a possible (developing) myelosuppression (to be determined by investigator or treating physician)
    - Triglycerides > 9 mmol/l
- Intercurrent severe illness or major surgery
- Protocol violations or if the requirements of the protocol are not respected
- Patient lost to follow up

8.5 Replacement of individual subjects after withdrawal

Subjects will not be replaced after withdrawal.

8.6 Follow-up of subjects withdrawn from treatment

Subjects withdrawn from treatment will continue to be treated at our center, outside the study. All efforts will be made to report the observations for the reason(s) for premature withdrawal and the time of occurrence. If an adverse event is the reason for withdrawal, the physicians of our department will administer therapy as clinically indicated. All serious adverse events and those adverse events for which the relationship to the study drug is plausibly related will be followed up until they have returned to baseline status or stabilized.

8.7 Premature termination of the study

Alitretinoin is registered for the treatment of hand eczema. Azathioprine has been used for decades to treat hand eczema and atopic dermatitis, among other dermatoses. Therefore we do not expect a substantial number of unexpected serious adverse events.
If an unforeseen substantial amount of (unexpected) serious adverse events occur, we will consider premature termination of the study.

9. SAFETY REPORTING

9.1 Section 10 WMO event

In accordance to section 10, subsection 1, of the WMO, the investigator will inform the subjects and the reviewing accredited METC if anything occurs, on the basis of which it appears that the disadvantages of participation may be significantly greater than was foreseen in the research proposal. The study will be suspended pending further review by the accredited METC, except insofar as suspension would jeopardise the subjects' health. The investigator will keep all subjects informed.

9.2 AEs, SAEs and SUSARs

9.2.1 Adverse events (AEs)

Adverse events are defined as any undesirable experience occurring to a subject during the study, whether or not considered related to the investigational product. All adverse events reported spontaneously by the subject or observed by the investigator or his staff will be recorded.

9.2.2 Serious adverse events (SAEs)

A serious adverse event is any untoward medical occurrence or effect at any dose that:

- results in death;
- is life threatening (at the time of the event);
- requires hospitalisation or prolongation of existing inpatients' hospitalisation;
- results in persistent or significant disability or incapacity;
- is a congenital anomaly or birth defect;

Any other important medical event that may not result in death, be life threatening, or require hospitalization, may be considered a serious adverse experience when, based upon appropriate medical judgement, the event may jeopardize the subject or may require an intervention to prevent one of the outcomes listed above.

The investigator will report the SAEs through the web portal *ToetsingOnline* to the accredited METC that approved the protocol, within 15 days after the investigator has first knowledge of the serious adverse events.

SAEs that result in death or are life threatening should be reported expedited. The expedited reporting will occur not later than 7 days after the responsible investigator has first knowledge of the adverse event. This is for a preliminary report with another 8 days for completion of the report.

9.2.3 Suspected unexpected serious adverse reactions (SUSARs)

Adverse reactions are all untoward and unintended responses to an investigational product related to any dose administered.

Unexpected adverse reactions are SUSARs if the following three conditions are met:

1. the event must be serious (see chapter 9.2.2);
2. there must be a certain degree of probability that the event is a harmful and an undesirable reaction to the medicinal product under investigation, regardless of the administered dose;
3. the adverse reaction must be unexpected, that is to say, the nature and severity of the adverse reaction are not in agreement with the product information as recorded in:
    - Summary of Product Characteristics (SPC) for an authorised medicinal product;
    - Investigator’s Brochure for an unauthorised medicinal product.

The investigator will report expedited the following SUSARs through the web portal *ToetsingOnline* to the METC:

- SUSARs that have arisen in the clinical trial that was assessed by the METC;
- SUSARs that have arisen in other clinical trials of the same investigator and with the same medicinal product, and that could have consequences for the safety of the subjects involved in the clinical trial that was assessed by the METC.

The remaining SUSARs are recorded in an overview list (line-listing) that will be submitted once every half year to the METC. This line-listing provides an overview of all SUSARs from the study medicine, accompanied by a brief report highlighting the main points of concern.
The expedited reporting of SUSARs through the web portal ToetsingOnline is sufficient as notification to the competent authority.

The investigator will report expedited all SUSARs to the competent authorities in other Member States, according to the requirements of the Member States.

The expedited reporting will occur not later than 15 days after the investigator has first knowledge of the adverse reactions. For fatal or life threatening cases the term will be maximal 7 days for a preliminary report with another 8 days for completion of the report.

Since the treating physician is not blinded, there is no need to break the code in case of an SUSAR.

9.3 Annual safety report

In addition to the expedited reporting of SUSARs, the investigator will submit, once a year throughout the clinical trial, a safety report to the accredited METC, competent authority, and competent authorities of the concerned Member States.

This safety report consists of:

- a list of all suspected (unexpected or expected) serious adverse reactions, along with an aggregated summary table of all reported serious adverse reactions, ordered by organ system, per study;
- a report concerning the safety of the subjects, consisting of a complete safety analysis and an evaluation of the balance between the efficacy and the harmfulness of the medicine under investigation.

9.4 Follow-up of adverse events

All AEs will be followed until they have abated, or until a stable situation has been reached. Depending on the event, follow up may require additional tests or medical procedures as indicated, and/or referral to the general physician or a medical specialist. SAEs need to be reported till end of study within the Netherlands, as defined in the protocol.

9.5 Data Safety Monitoring Board (DSMB)

No DSMB is established, since this is a daily practice study. In case of life-threatening diseases usually the implementation of a DSMB is indicated from an ethical point of view. But hand eczema is a non-critical indication. Frequent laboratory assessments will reduce the possibility of serious adverse events to a minimum. The patient population in this clinical trial exists of legal competent adults and the study drugs alitretinoin and azathioprine are well-investigated, well-characterized drugs.

10. STATISTICAL ANALYSIS

This trial is designed to demonstrate a superior response to azathioprine compared to alitretinoin in the treatment of severe chronic non-hyperkeratotic hand eczema. Response to treatment is defined as an improvement of ≥ 2 steps on the Physician Global Assessment (PGA) developed by Ruzicka et al (8,9) at 24 weeks of treatment.

All analysis will be based on the intention-to-treat principle to guard against attrition bias. Subjects might not only withdraw because the study drug works insufficient, they might also withdraw when their hand eczema is cured.

Missing values will be handled in a way that is dependent on assumptions about the missing data. If the extent and pattern of missing data is known (e.g. missing at random (MAR), missing completely at random (MCAR), missing not at random (MNAR)), an analysis will be chosen that is valid under a plausible assumption about the missing data (probably mixed models). This is according to a strategy proposed by White et al. (28)

10.1 Primary study parameter

Severity of hand eczema

Between-group difference in response to treatment between baseline and 24 weeks of treatment. For comparison of proportions in the alitretinoin and azathioprine group the chi-square test, or Fisher's exact test if appropriate, will be used.

10.2 Secondary study parameters

Severity of hand eczema

- Between-group difference in mean change in PGA between baseline and 12 and 24 weeks. For comparison of mean change between the alitretinoin and azathioprine group at week 12 and 24, the Student's t-test or Mann-Whitney U-test will be used, depending on distribution of data.
- Between-group difference in response to treatment between baseline and 12 weeks of treatment. For comparison of proportions in the alitretinoin and azathioprine group the chi-square test will be used.
- Between-group difference in mean change between baseline and week 4, 8, 12 and 24, assessed by the Hand Eczema Severity Index (HECSI) score. This will be described graphically. For comparison of mean change between the

alitretinoin and azathioprine group at week 12 and 24, the Student's t-test or Mann-Whitney U-test will be used, depending on distribution of data.

- Between-group difference in time to response (time to first PGA improvement of ≥ 2 steps). This will be analysed using a Kaplan-Meier product limit estimator. For comparison between the alitretinoin and azathioprine group the Log-rank test will be used.

Patient rated outcome measures (PROMs):
Quality of life

- Between-group mean change in quality of life between baseline and 12 and 24 weeks, assessed by the Quality Of Life in Hand Eczema Questionnaire (QOLHEQ). Clinically relevant improvement is defined as an absolute improvement of 15 points (theoretically corresponding to an improvement of ≥ 1 point on 50% of the questions) compared to baseline. For comparison of proportions of patients rated as having clinically relevant improvement in the alitretinoin and azathioprine group, the chi-square test will be used.

Patient reported improvement

- Between-group difference in patients reporting improvement as 'clear or almost clear' at week 12 and 24, assessed by Patient Global Assessment (PaGA). For comparison of proportions of patients rated as 'clear or almost clear' in the alitretinoin and azathioprine group, the chi-square test will be used.

Safety and tolerability

- Adverse events in both groups will be registered.

Cost-utility and cost-effectiveness

- For each group (alitretinoin and cyclosporine), the mean EQ-5D scores overall and of each dimension will be reported. Results from the descriptive system of the EQ-5D-5L will be converted to a utility index value, a population based (social) value specific for the Netherlands. With this value, Dutch utility values will be calculated in order to determine the quality adjusted life years (QALYs) over the study period. Mean values of the EQ-VAS will be reported with a 95% confidence interval. For comparison of means, the Student's t-test or Mann-Whitney U-test will be used, depending on distribution of data.
- The incremental cost-effectiveness ratio (ICER) will be calculated and reported as €/QALY. Effect will be assessed with the PGA. Costs will be assessed as described in paragraph 8.2.

- A regression model will be used to estimate the association between QALYs and the PGA.

10.3 Other study parameters

Continuous data with normal distribution will be presented as mean ± SD. Variables with a skewed distribution will be presented as median and range. Categorical variables will be expressed as frequencies and percentages. Confidence intervals (CIs) are expressed to a level of 95%.
To test for normal distribution of data, the data will be visually inspected, a Q-Q plot will be constructed and in case of doubt a test for normality will be used (e.g. Shapiro-Wilk test/Kolmogorov-Smirnov test).
For comparing normally distributed continuous data, we will use the Student’s t-test. In variables with non-homogeneity of variance, the Welch test will be used.
For comparing non-normally distributed continuous data, the Mann-Whitney U test will be used.
For comparing categorical data the chi-square test will be used and the Fisher’s exact test will be used in variables containing low numbers.
All P-values are two-sided and a P-value < 0,05 is considered statistically significant.
Statistical analysis will be performed using SPSS 22 (SPSS Inc, Chicago, IL, USA).

10.4 Interim analysis

Not applicable.

11. ETHICAL CONSIDERATIONS

11.1 Regulation statement

This study will be conducted according to the principles of the Declaration of Helsinki (Seventh revision, Fortaleza 2013), in accordance with the Medical Research Involving Human Subjects Act (WMO), and also in accordance with the International Conference on Harmonisation Good Clinical Practice Guidelines.

11.2 Recruitment and consent

It is the responsibility of the investigator, or a person designated by the investigator, to obtain written informed consent from each individual participating in this study after adequate explanation of the aims, methods, objectives and potential hazards of the study. It will also be explained to patients that they are completely free to refuse to enter the study or to withdraw from it at any time for any reason. Patients will be given a period of one week to consider participation before they are asked to sign the informed consent form.

If new safety information results in significant changes in the risk/benefit assessment, the consent form will be reviewed and updated if necessary. All patients (including those already being treated) will be informed of the new information, given a copy of the revised form and asked to give their consent to continue in the study.

The patient information letter and informed consent form are attached as separate documents.

11.3 Objection by minors or incapacitated subjects

Not applicable.

11.4 Benefits and risks assessment, group relatedness

In this trial both groups are treated with a drug, known to be beneficial to hand eczema in a considerable amount of patients. So the intended benefit of both study drugs is to reduce the severity of hand eczema.

We hypothesize that azathioprine has a superior efficacy compared to alitretinoin (the registered treatment for hand eczema in The Netherlands) in severe chronic non-hyperkeratotic hand eczema. If this hypothesis is confirmed, there could be a practical, as well as a financial implication. Practically, more responding patients to azathioprine leads to a greater beneficial effect on hand eczema in this patient group. Financially, azathioprine is a lot less expensive than alitretinoin. If azathioprine shows superior efficacy in non-hyperkeratotic hand eczema, this could lead to an official registration. This,

in turn, could mean a decrease in financial burden for the treatment of all non-hyperkeratotic hand eczema patients in the population.
A risk assessment is carried out in chapter 13.

11.5 Compensation for injury

The sponsor/investigator has a liability insurance which is in accordance with article 7, subsection 6 of the WMO.

The sponsor (also) has an insurance which is in accordance with the legal requirements in the Netherlands (Article 7 WMO and the Measure regarding Compulsory Insurance for Clinical Research in Humans of 23th June 2003). This insurance provides cover for damage to research subjects through injury or death caused by the study.

1. € 450.000,-- (i.e. four hundred and fifty thousand Euro) for death or injury for each subject who participates in the Research;
2. € 3.500.000,-- (i.e. three million five hundred thousand Euro) for death or injury for all subjects who participate in the Research;
3. € 5.000.000,-- (i.e. five million Euro) for the total damage incurred by the organisation for all damage disclosed by scientific research for the Sponsor as ‘verrichter’ in the meaning of said Act in each year of insurance coverage.

The insurance applies to the damage that becomes apparent during the study or within 4 years after the end of the study.

11.6 Incentives

Not applicable.

12. ADMINISTRATIVE ASPECTS, MONITORING AND PUBLICATION

12.1 Handling and storage of data and documents

Data will be handled confidentially. Data derived from the questionnaires and other paper source documents will be coded using sequential administration numbers. A subject identification code list is used to link the data to the subjects. The code is not based on the patient initials and birth-date. The code will be safeguarded by the principal investigator, dr. M.L.A. Schuttelaar. The documents will be stored in a locked room.
The digital source data will be saved in subsections of the subjects medical file. These data will be accessible to the principal investigator and the investigator, and also to the treating physician if this is not the investigator. Data will not be accessed by the blinded efficacy assessors.
All data will be recorded in electronic Case Report Forms in Utopia, software for Electronic Data Capture (, developed by the Trial Coordination Center, linked to the University Medical Center Groningen. The eCRFs will only be accessible with the username and password of the responsible investigator.
Data will be saved for 15 years after completion of this study.
All the data will be saved in accordance with the Dutch Personal Data Protection Act.

12.2 Monitoring and Quality Assurance

A monitor of the University Medical Center Groningen will carry out monitoring of this study. The monitor will get read-only access to the digital and paper documents of participants. Goal of this monitoring is to review if:

- the rights and wellbeing of subjects are being protected
- the reported data is right and fully reproducible
- the execution of the study is in accordance with this protocol and relevant legal requirements.

12.3 Amendments

A ‘substantial amendment’ is defined as an amendment to the terms of the METC application, or to the protocol or any other supporting documentation, that is likely to affect to a significant degree:

- the safety or physical or mental integrity of the subjects of the trial;
- the scientific value of the trial;
- the conduct or management of the trial; or
- the quality or safety of any intervention used in the trial.

All substantial amendments will be notified to the METC and to the competent authority.

Non-substantial amendments will not be notified to the accredited METC and the competent authority, but will be recorded and filed by the investigator.

12.4 Annual progress report

The investigator will submit a summary of the progress of the trial to the accredited METC once a year. Information will be provided on the date of inclusion of the first subject, numbers of subjects included and numbers of subjects that have completed the trial, serious adverse events/ serious adverse reactions, other problems, and amendments.

12.5 End of study report

The investigator will notify the accredited METC and the competent authority of the end of the study within a period of 90 days. The end of the study is defined as the last patient's last visit.

In case the study is ended prematurely, the investigator will notify the accredited METC and the competent authority within 15 days, including the reasons for the premature termination.

Within one year after the end of the study, the investigator will submit a final study report with the results of the study, including any publications/abstracts of the study, to the accredited METC and the Competent Authority.

12.6 Public disclosure and publication policy

This study is designed honoring the CCMO statement on publication policy.
This is an investigator initiated study. The results of the study will be made public unreservedly; they will be offered for publication in a peer reviewed journal. In a publication all data will be handled anonymously.
The study will be registered in a public trail register before the inclusion of the first patient.

13. STRUCTURED RISK ANALYSIS

13.1 Potential issues of concern

Both alitretinoin and azathioprine are drugs that are routinely used in medicine and particularly in the treatment of hand eczema in our department. The information asked in this chapter is described in detail in the SPC text documents of the study drugs. (10,18) These texts are used as important sources on which this protocol is based.

a. Level of knowledge about mechanism of action

For this section the reader is referred to paragraph 6.1 and the SPC text of alitretinoin and azathioprine. (10,18)

b. Previous exposure of human beings with the test product(s) and/or products with a similar biological mechanism

For this section the reader is referred to paragraph 6.3 and the SPC text of alitretinoin and azathioprine. (10,18)

c. Can the primary or secondary mechanism be induced in animals and/or in *ex-vivo* human cell material?

For this section the reader is referred to paragraph 6.2 and the SPC text of alitretinoin and azathioprine. (10,18)

d. Selectivity of the mechanism to target tissue in animals and/or human beings

For this section the reader is referred to paragraph 6.1 and the SPC text of alitretinoin and azathioprine. (10,18)

e. Analysis of potential effect

Alitretinoin (from SPC text) (10)

The recommended dose range for alitretinoin is 10 mg - 30 mg once daily.

The recommended start dose for alitretinoin is 30 mg once daily. A dose reduction to 10 mg once daily may be considered in patients with unacceptable adverse reactions to the higher dose. In studies investigating 10 mg and 30 mg daily doses, both doses resulted in clearing of the disease. The 30 mg dose provided a more rapid response and a higher response rate. The 10 mg daily dose was associated with fewer adverse events.

A treatment course of alitretinoin may be given for 12 to 24 weeks depending on response. Discontinuation of therapy should be considered for patients who still have severe disease after the initial 12 weeks of treatment. In the event of relapse, patients may benefit from further treatment courses of alitretinoin.

The capsules should be taken with a meal once daily.
Alitretinoin should not be prescribed if the patient's eczema can be adequately controlled by standard measures, including skin protection, avoidance of allergens and irritants, and treatment with potent topical corticosteroids.
Risks of alitretinoin treatment are described in paragraph 6.4.

Azathioprine (from SPC text) (18)
In general, the starting dosage is 1-3mg/kg/body weight/day and should be adjusted according to the clinical response (which may not be evident for weeks or months) and haematological tolerance.
The maintenance dosage required may range from less than 1mg/kg body weight/day to 3mg/kg/body weight/day depending on the clinical condition being treated and the individual patient response including haematological tolerance.
Risks of azathioprine treatment are described in paragraph 6.4 and 6.5 (regarding TPMT measurement).

<u>f. Pharmacokinetic considerations</u>
Alitretinoin (from SPC text) (10)
Absorption
The absorption of alitretinoin from the gastro-intestinal tract is variable and dose-proportional over the therapeutic range from 10-30 mg. The absolute bioavailability of alitretinoin has not been determined. When alitretinoin is taken with food, the systemic exposure is enhanced by a factor of 4 and the variability of exposure is decreased. Therefore, alitretinoin should be taken with a meal.

Distribution
Alitretinoin strongly binds to plasma proteins. The volume of distribution of alitretinoin in man has not been determined, but animal studies indicate a volume of distribution greater than the extracellular volume.

Metabolism
Alitretinoin is metabolized by oxidation in the liver by CYP3A4 isoenzymes into 4-oxo-alitretinoin. Both compounds undergo isomerization into all-trans retinoic acid and 4-oxo-all-trans retinoic acid. After oral administration, the contribution of the metabolites in plasma to the systemic exposure of alitretinoin is approximately 35% to 80% for 4-oxo-alitretinoin. The major metabolite 4-oxo-alitretinoin is further glucuronidated and eliminated in urine. Alitretinoin is degraded similarly to vitamin A by sequential cleavage of

the carbon-side chain. During a 12-to 24-week treatment period with 10 or 30 mg dose, the exposure to alitretinoin remained stable.

Elimination

Alitretinoin is an endogenous retinoid. Alitretinoin concentrations return to normal range within 1 to 3 days treatment cessation. Excretion of radio-labelled alitretinoin was complete with approximately 94% of the dose recovered. Radio-labelled material was eliminated mainly in urine and a smaller fraction (approx. 30%) in faeces. The most abundant excretion compound is the glucuronide of 4-oxo-alitretinoin amounting to 6.5% of the dose in urine. Elimination half-life of unchanged alitretinoin ranges between 2 to 10 hours. Alitretinoin and its 4-oxo-metabolite do not accumulate.

Pharmacokinetic in special populations

In a pharmacokinetic study in patients, gender, weight and age did not affect the pharmacokinetics of alitretinoin. The pharmacokinetics of alitretinoin in CHE patients was similar to that in healthy volunteers. Alitretinoin kinetics has not been studied in patients with hepatic or with severe renal insufficiency or in patients below 18 years.

Azathioprine (from SPC text) (18)

Azathioprine is well absorbed following oral administration. Peak plasma concentrations are reached 1-2 hours after taking a dose. Azathioprine is distributed rapidly throughout the body. The plasma half life is 3-5 hours. Only 30% of the medicinal product binds to plasma proteins. 12.5% enter the cerebrospinal fluid.

Azathioprine is extensively metabolised to 6-thioinosinic acid and methyl mercaptopurine-ribonucleotide, which, in part, are responsible for the effect of the medicinal product.

The effect in-vivo is complicated by the action of methyl-nitroimidazole, which is also found.

Up to 50% of a dose is excreted in urine during the first 24 hours after administration, with approximately 10% as unchanged substance. Only 12.6% of the dose is excreted during 48 hours with the faeces. There is no evidence for enterohepatic circulation.

A lowered dosage for patients with reduced renal function may be necessary, probably as a result of reduced elimination of the active metabolites of azathioprine.

Also in patients with hepatic impairment the metabolism of azathioprine is altered. Conversion into the active form is reduced, and especially the breakdown to eliminable metabolites is diminished.

Mercaptopurine, a metabolite of azathioprine, has been identified in the colostrum and breast-milk of women receiving azathioprine treatment.

g. Study population

In general, otherwise healthy subject with severe, chronic non-hyperkeratotic hand eczema will be included. Woman with child bearing potential will only be included in the study if they agree to use proper contraceptive methods during study period and up to two months following end of study.

h. Interaction with other products

For this section the reader is referred to paragraph 5.2 and the SPC text of alitretinoin and azathioprine. (10,18)

i. Predictability of effect

No biomarkers for effect have been determined.

j. Can effects be managed?

Alitretinoin (from SPC text) (10)

Alitretinoin is a derivative of vitamin A. Alitretinoin has been administered in oncological clinical studies at dosages of more than 10-times of the therapeutic dosage given for chronic hand eczema. The adverse effects observed were consistent with retinoid toxicity, and included severe headache, diarrhoea, facial flushing, hypertriglyceridemia. These effects were reversible.

It can be concluded that the (reversible) effects can be properly managed.

Azathioprine (from SPC text) (18)

Symptoms:

In the event of overdose the most likely effect is bone marrow suppression, reaching its maximum mostly 9-19 days after dosing. The principal signs of bone marrow suppression are ulceration of the throat, fever and infections. Furthermore, bruising, bleeding and fatigue may occur. A single large dose of azathioprine is less likely to have a toxic effect than a chronic minor overdosage (e.g. on prescription). Although improvement may be delayed, it usually occurs from the twelfth day after overdose, provided that the patient has not taken a high dose in the meantime.

Treatment:

There is no specific antidote for azathioprine. In the event of overdose, blood count and hepatic function in particular should be monitored. Azathioprine is known to be dialysable and in severe cases dialysis may be used.

It can be concluded that management of severe effects, if needed, could be radical but possible.

13.2 Synthesis

The overall risks for patients participating in this study are acceptable because of the tight inclusion and exclusion criteria (ensuring a relatively healthy study population), combined with regular laboratory assessments to enhance safety monitoring by the investigator or treating physician. Furthermore, prior experience with both study drugs in daily practice has improved our capability to manage risks.

A specific measure that will be taken to reduce risks is the TPMT measurement. This will be carried out in the azathioprine group to exclude patients with a low or absent TPMT activity, to prevent certain life-threatening myelumsuppression to occur in these patients.

In general, adult subjects with severe chronic non-hyperkeratotic hand eczema will be included, who are otherwise healthy. The remaining risk is therefore small, does not differ considerably from regular daily practice, and is therefore deemed acceptable for study subjects.

14. REFERENCES

(1) Thyssen JP, Johansen JD, Linneberg A, Menné T. The epidemiology of hand eczema in the general population--prevalence and main findings. Contact Dermatitis 2010 Feb;62(2):75-87.

(2) Mortz CG, Bindslev-Jensen C, Andersen KE. Hand eczema in The Odense Adolescence Cohort Study on Atopic Diseases and Dermatitis (TOACS): prevalence, incidence and risk factors from adolescence to adulthood. Br J Dermatol 2014 Aug;171(2):313-323.

(3) Diepgen TL, Elsner P, Schliemann S, Fartasch M, Köllner A, Skudlik C, et al. Guideline on the management of hand eczema ICD-10 Code: L20. L23. L24. L25. L30. J Dtsch Dermatol Ges 2009 May;7 Suppl 3:S1-16.

(4) English J, Aldridge R, Gawkrodger DJ, Kownacki S, Statham B, White JM, et al. Consensus statement on the management of chronic hand eczema. Clin Exp Dermatol 2009 Oct;34(7):761-769.

(5) Lynde C, Guenther L, Diepgen TL, Sasseville D, Poulin Y, Gulliver W, et al. Canadian hand dermatitis management guidelines. J Cutan Med Surg 2010;14(6):267-84.

(6) Menné T, Johansen JD, Sommerlund M, Veien NK, Danish Contact Dermatitis Group. Hand eczema guidelines based on the Danish guidelines for the diagnosis and treatment of hand eczema. Contact Dermatitis 2011 Jul;65(1):3-12.

(7) Coenraads PJ, Christoffers WA, Svensson Å, Diepgen TL, Blok JL, Matterne U, et al. Cochrane review: Interventions for hand eczema. Re-submitted to the Cochrane Database of Systematic Reviews June 2014 .

(8) Ruzicka T, Larsen FG, Galewicz D, Horváth A, Coenraads PJ, Thestrup-Pedersen K, et al. Oral alitretinoin (9-cis-retinoic acid) therapy for chronic hand dermatitis in patients refractory to standard therapy: results of a randomized, double-blind, placebo-controlled, multicenter trial. Arch Dermatol 2004 Dec;140(12):1453-1459.

(9) Ruzicka T, Lynde CW, Jemec GB, Diepgen T, Berth-Jones J, Coenraads PJ, et al. Efficacy and safety of oral alitretinoin (9-cis retinoic acid) in patients with severe chronic hand eczema refractory to topical corticosteroids: results of a randomized, double-blind, placebo-controlled, multicentre trial. Br J Dermatol 2008 Apr;158(4):808-817.

(10) SPC text alitretinoin. 2014; Available at: http://db.cbg-meb.nl/IB-teksten/h100957.pdf. Accessed February 03, 2015.

(11) Schmitt J. Ciclosporin versus alitretinoin for severe atopic hand dermatitis (TocyDD). 2013; Available at: clinicaltrials.gov/show/NCT01231854. Accessed January 06, 2015.

(12) Scerri L. Azathioprine in dermatological practice. An overview with special emphasis on its use in non-bullous inflammatory dermatoses. Adv Exp Med Biol 1999;455:343-348.

(13) Pearce VJ, Mortimer PS. Hand dermatitis and lymphoedema. Br J Dermatol 2009 Jul;161(1):177-180.

(14) Agarwal US, Besarwal RK. Topical clobetasol propionate 0.05% cream alone and in combination with azathioprine in patients with chronic hand eczema: an observer blinded

randomized comparative trial. Indian J Dermatol Venereol Leprol 2013 Jan-Feb;79(1):101-103.

(15) Schram ME, Roekevisch E, Leeflang MM, Bos JD, Schmitt J, Spuls PI. A randomized trial of methotrexate versus azathioprine for severe atopic eczema. J Allergy Clin Immunol 2011 Aug;128(2):353-359.

(16) Coenraads PJ, Van Der Walle H, Thestrup-Pedersen K, Ruzicka T, Dreno B, De La Loge C, et al. Construction and validation of a photographic guide for assessing severity of chronic hand dermatitis. Br J Dermatol 2005 Feb;152(2):296-301.

(17) Long CC, Finlay AY. The finger-tip unit--a new practical measure. Clin Exp Dermatol 1991 Nov;16(6):444-447.

(18) SPC text azathioprine. 2014; Available at: http://db.cbg-meb.nl/IB-teksten/h05565.pdf. Accessed February 04, 2015.

(19) Bissonnette R, Worm M, Gerlach B, Guenther L, Cambazard F, Ruzicka T, et al. Successful retreatment with alitretinoin in patients with relapsed chronic hand eczema. Br J Dermatol 2010 Feb 1;162(2):420-426.

(20) Meggitt SJ, Anstey AV, Mohd Mustapa MF, Reynolds NJ, Wakelin S. British Association of Dermatologists' guidelines for the safe and effective prescribing of azathioprine 2011. Br J Dermatol 2011 Oct;165(4):711-734.

(21) Held E, Skoet R, Johansen JD, Agner T. The hand eczema severity index (HECSI): a scoring system for clinical assessment of hand eczema. A study of inter- and intraobserver reliability. Br J Dermatol 2005 Feb;152(2):302-307.

(22) Hakkaart-van Roijen L, Tan S, Bouwmans C. Handleiding voor kostenonderzoek, methoden en standaard kostprijzen voor economische evaluaties in de gezondheidszorg. College voor zorgverzekeringen. Geactualiseerde versie 2010.

(23) Williams HC, Burney PG, Pembroke AC, Hay RJ. The U.K. Working Party's Diagnostic Criteria for Atopic Dermatitis. III. Independent hospital validation. Br J Dermatol 1994 Sep;131(3):406-416.

(24) Lukács J, Schliemann S, Elsner P. Association between smoking and hand dermatitis - a systematic review and meta-analysis. J Eur Acad Dermatol Venereol 2015 Feb 4.

(25) Weintraub WS, Klein LW, Seelaus PA, Agarwal JB, Helfant RH. Importance of total life consumption of cigarettes as a risk factor for coronary artery disease. Am J Cardiol 1985 Mar 1;55(6):669-672.

(26) Masters N, Tutt C. Smoking Pack Years Calculator [Internet]. 2007 May 21; Available at: http://www.smokingpackyears.com. Accessed July 17, 2015.

(27) Ofenloch RF, Weisshaar E, Dumke AK, Molin S, Diepgen TL, Apfelbacher C. The Quality of Life in Hand Eczema Questionnaire (QOLHEQ): validation of the German version of a new disease-specific measure of quality of life for patients with hand eczema. Br J Dermatol 2014 Aug;171(2):304-312.

(28) White IR, Horton NJ, Carpenter J, Pocock SJ. Strategy for intention to treat analysis in randomised trials with missing outcome data. BMJ 2011 Feb 7;342:d40.
